# *AKT1* and *SELP* Polymorphisms Predict the Risk of Developing Cachexia in Pancreatic Cancer Patients

**DOI:** 10.1371/journal.pone.0108057

**Published:** 2014-09-19

**Authors:** Abolfazl Avan, Amir Avan, Tessa Y. S. Le Large, Andrea Mambrini, Niccola Funel, Mina Maftouh, Majid Ghayour-Mobarhan, Maurizio Cantore, Ugo Boggi, Godefridus J. Peters, Paola Pacetti, Elisa Giovannetti

**Affiliations:** 1 Department of Medical Oncology, VU University Medical Center, Amsterdam, The Netherlands; 2 Biochemistry of Nutrition Research Center, and Department of New Sciences and Technology, School of Medicine, Mashhad University of Medical Sciences, Mashhad, Iran; 3 Department of Medical Oncology, Carrara Civic Hospital, Carrara, Italy; 4 Start-Up Unit, University of Pisa, Pisa, Italy; Centro Nacional de Investigaciones Oncológicas (CNIO), Spain

## Abstract

Pancreatic ductal adenocarcinoma (PDAC) patients have the highest risk of developing cachexia, which is a direct cause of reduced quality of life and shorter survival. Novel biomarkers to identify patients at risk of cachexia are needed and might have a substantial impact on clinical management. Here we investigated the prognostic value and association of *SELP-rs6136*, *IL6-rs1800796* and *AKT1-rs1130233* polymorphisms with cachexia in PDAC. Genotyping was performed in DNA from blood samples of a test and validation cohorts of 151 and 152 chemo-naive locally-advanced/metastatic PDAC patients, respectively. The association of *SELP-rs6136*, *IL6-rs1800796* and *AKT1-rs1130233* polymorphisms with cachexia as well as the correlation between cachexia and the candidate polymorphisms and overall survival were analyzed. Akt expression and phosphorylation in muscle biopsies were evaluated by specific ELISA assays. *SELP-rs6136-AA* and *AKT1-rs1130233-AA/GA* genotypes were associated with increased risk of developing cachexia in both cohorts (*SELP: p* = 0.011 and *p* = 0.045; *AKT1: p* = 0.004 and *p* = 0.019 for the first and second cohorts, respectively), while patients carrying *AKT1-rs1130233-GG* survived significantly longer (*p* = 0.002 and *p* = 0.004 for the first and second cohorts, respectively). In the multivariate analysis *AKT1-rs1130233-AA/GA* genotypes were significant predictors for shorter survival, with an increased risk of death of 1.7 (*p* = 0.002) and 1.6 (*p* = 0.004), in the first and second cohorts, respectively. This might be explained by the reduced phosphorylation of Akt1 in muscle biopsies from patients harboring *AKT1-rs1130233-AA/GA* (*p* = 0.003), favoring apoptosis induction. In conclusion, *SELP* and *AKT1* polymorphisms may play a role in the risk of cachexia and death in PDAC patients, and should be further evaluated in larger prospective studies.

## Introduction

Cachexia is a multi-factorial, systemic syndrome characterized by pathological wasting of skeletal muscle and adipose tissue mass that leads to pronounced weight loss. It can occur in the course of several chronic illnesses, but it is most frequently observed concomitantly with malignancies [1]. In particular, patients with pancreatic ductal adenocarcinoma (PDAC) have the highest prevalence of cachexia, and often experience the most severe symptoms of this disease [2]. Recent studies showed that existing preoperative cachexia reduces the post-operative outcome of PDAC patients [3], [4]. In advanced PDAC the presence of cachexia is also associated with a worse prognosis, and stabilizing weight can be crucial to prolong survival [5]. Moreover, cachexia decreases the tolerance to systemic treatments and dramatically affects the quality of life [6].

Extensive preclinical and clinical studies have evaluated the underlying mechanisms of cachexia in PDAC, revealing a complex of multiple interdependent patient- and cancer-specific components. Several hormones and tumor-derived factors can contribute to tissue catabolism and appetite regulation, leading to anorexia and fatigue, whereas symptoms such as chronic pain, nausea and pancreatic insufficiency, as well as adverse effects of anticancer therapies, might further reduce appetite and nutritional intake [7].

However, in contrast to cachectic patients with chronic pancreatitis, PDAC patients with cachexia have significantly reduced hemoglobin and albumin levels, associated to the systemic reaction to both the tumor and the inflammatory processes [4]. Specific pro-inflammatory cytokines such as interleukin 1-beta (IL-1β), interleukin 6 (IL-6), tumor necrosis factor alpha (TNFα), and interferon gamma have been shown to be associated with progressive weight loss, through the acute phase protein response in the liver [8]–[10]. This systemic inflammation can also convey its action via local inflammatory signaling, activated by the nuclear transcription factor kB (NF-kB), and in both mouse models and muscle biopsy specimens NF-kB–mediated Pax7 dysregulation emerged among the causes of muscle wasting in PDAC [11]. Furthermore, in cachexia the balance between the anabolism and the catabolism of proteins is tipped toward a catabolic state resulting from activated ubiquitin proteasome and autophagy systems that promote protein breakdown, as well as from reduced Akt activity, that decreases protein synthesis [1], [12]. The regulation of muscle differentiation is indeed dependent on the activation of signal transduction cascades with the complex involvement of key kinases, such as the serine/threonine kinase Akt, and previous studies demonstrated that Akt is essential to promote protein synthesis and cell survival and to block protein degradation [13], All this evidence supports its biological relevance in the context of cachexia.

Although the variable production of the pro-inflammatory cytokines, NF-kB activation and Akt phosphorylation status depends on a variety of clinical and pathological factors, there is growing evidence that genetic variations might also affect these key determinants of cachexia. In particular, the *G-*allele of the *IL6-rs1800796* polymorphism was associated with decreased survival and increased susceptibility to cachexia in Chinese PDAC patients [14]. Moreover, in a systematic study on genetic determinants of cachexia, out of 92 potential candidate genes with 184 polymorphisms relating to cancer cachexia, 42 polymorphisms across 33 genes have been identified for having a functional or clinical relevance in more than one study [15]. Of these 42 polymorphisms, 13 had more than one effect on clinical features associated with cancer cachexia (i.e. inflammation, loss of fat mass and/or lean mass, and reduced survival). Further pathway analysis of these candidates revealed 4 genes (*ADIPOQ*, *IL6*, *NFKB1* and *TLR4*) interlinking two putative major networks involved in the development of cancer cachexia. However, in a recent study using these selected polymorphisms only the *C-*allele of the single nucleotide polymorphism (SNP) *rs6136*-*SELP* was found to be associated with weight loss of >10%, both in the first study cohort of 775, including 114 PDAC patients, and in the validation cohort of 101 cancer patients, including 6 PDAC patients [16]. The *SELP* gene encodes a 140 kDa protein stored in the alpha-granules of platelets and Weibel-Palade bodies of endothelial cells, which redistributes to the plasma membrane during platelet activation and degranulation and functions as a cell adhesion molecule (CAM) mediating the interaction of activated endothelial cells or platelets with leukocytes during the initial steps in inflammation [17].

However, the largest study on cancer cachectic patients (N = 1797), including 35 PDAC patients, failed to validate the predictive role of 9 candidate SNPs [18]. One of the possible explanations for not being able to confirm genetic associations may be the gene–environment relations specific for the studied disease. Previous analysis supported the role of the *IL6-rs1800796* and *SELP-rs6136* SNPs as susceptibility biomarkers for PDAC cachexia [13], [15], while our recent studies revealed a key functional role of the *AKT1-rs1130233* SNP [19]. Therefore in the present study we evaluated the association of these candidate polymorphisms in the *AKT1*, *IL6*, and *SELP* genes with cachexia in a total population of 303 PDAC patients, in two independent cohorts.

## Materials and Methods

### Cachexia phenotype definition

To ensure that patients with or without cachexia were clearly distinguished, we used the “2011 International Consensus,” according to which cachexia is defined by unintended weight loss of more than 5% of body weight or weight loss of more than 2% in already depleted individuals with a body mass index (BMI) less than 20 kg/m^2^ over 6 months [20].

### Patients

In the current study we selected a first cohort of 151 patients, enrolled between 31st March 2004 and 10th January 2009, and a second/validation cohort of 152 patients, enrolled between 15th January 2009 and 15th January 2013. All the eligible patients were chemo-naive patients with diagnosis of histologically confirmed locally advanced or metastatic PDAC, treated at the Carrara Civic-Hospital (Carrara, Italy).

### Ethics

All the patients gave their written informed consent to the sample collection and analysis, and the study has received approval from the Ethics Committee of Carrara Civic-Hospital (Carrara, Italy) and Ethics Committee of Pisa University Hospital (Pisa, Italy) as a follow-up study of the research protocol entitled “Pharmacogenetics of gemcitabine-related genes in pancreas cancer: correlation with clinical outcome and tolerability”. The responsible investigators ensure that this study was conducted according to the Declaration of Helsinki, the European Guidelines on Good Clinical Practice, and relevant national and regional authority requirements.

### Genotyping

Genomic DNA was extracted from blood samples at the Laboratory Medical Oncology (VUmc, Amsterdam, The Netherlands) using the QIAamp DNA Mini-Kit according to the manufacturer protocol (Qiagen, San Diego, CA). The concentration and purity of DNAs was determined with the NanoDrop-1000-Detector (NanoDrop-Technologies, Wilmington, USA). Genotype analysis of *rs1800796*, *rs6136*, and *rs1130233* polymorphisms was performed using Taqman-based PCR reactions carried out in 12.5 µl total volume, using 20 ng of DNA diluted in TaqMan Universal Master Mix with specific primers and probes (SNP Genotyping Assays products C__11326893_10, C__11975277_20, and C__7489835_10, respectively). The ABIPRISM-7500 instrument (Applied Biosystems, Life Technologies, Foster City, CA) equipped with the SDS version-2.0 software was employed to evaluate the allelic content of each sample in the plate by reading the generated fluorescence.

### Analysis of Akt expression and phosphorylation in homogenized muscle biopsies

Previous studies evaluated the relationships of *AKT1* polymorphisms with AKT1 mRNA and protein expression in lymphoblastoids and lung cancer cells [19], [21], but no data are available on the correlation between *AKT1-rs1130233* and Akt1 protein expression and phosphorylation status in skeletal muscle. Therefore, we collected rectus abdominis muscle biopsies obtained during resection of primary PDAC. Small tissue pieces (about 100 mg) were immediately frozen in liquid nitrogen, using isopentane, after surgery at the University Hospital of Pisa (Pisa, Italy), according to a protocol approved by the local Hospital Ethic Committee. A total of 18 samples from chemonaive patients (nine for the *AKT1-rs1130233-AA/GA* and nine for the *AKT1-rs1130233-GG* genotype, as determined in preliminary genotyping analysis from blood samples, respectively) were evaluated.

The frozen tissues were homogenized using the micro-dismembrator, as described previously [22]. The frozen powder was extracted with 2.5% w/v sulfosalicyl acid and centrifuged (10 min, 8000 RPM) and the protein concentration in the extracts was determined using the Biorad protein assay (Life Science, Bio-Rad Laboratories B.V., Veenendaal, The Netherlands). Total Akt1 and Akt1 phosphorylation at serine-473 (Akt [pS473]) were evaluated with specific ELISA assays (Invitrogen, Life Technologies), and normalized to standard curves of Human-total-Akt1 and Human-phospho-Akt1, as well as to protein content, as described previously [23].

### Statistics

Demographic and clinical information were compared across genotype using Pearson's χ^2^ and logistic regression. In agreement with previous studies, the correlation with candidate genotypes was performed combining the less common homozygous and heterozygous genotypes [24].

To evaluate whether cachexia and the other clinical variables as well as the candidate polymorphisms affected clinical outcome, overall survival (OS) curves were analyzed from the day of treatment start to the end point (death or censoring) according to Kaplan–Meier method, and compared by log-rank. The significant prognostic variables in the univariate analysis were included in multivariate analysis, using Cox's proportional hazards model. This analysis included a step down procedure based on the likelihood ratio test, where hazard ratio (HR) was calculated to estimate the magnitude and the direction of the effect. Appropriate adjustment for false-positive report probability in the analysis of the polymorphisms was performed according to the Wacholder method [25].

All the analyses of the samples were done in a blinded fashion relative to clinical outcome. Data were analyzed using SPSS-20 software (IBM, IL, USA). All the analyses were two-sided and statistical significance was set at *p*-value of <0.05.

## Results

### Clinical characteristics and outcome

Patient baseline characteristics and their association with clinical outcome are summarized in Table 1. OS data were available from all patients.

**Table 1:** Clinical outcome according to baseline characteristics.

	First cohort			Second/validation cohort		
Characteristics	Patients, n (%)	OS median mo. (95% CI)	*p*-value*	Patients, n (%)	OS median mo. (95% CI)	*p*-value*
**No. patients**	151	12.5 (10.9–14.1)		152	12.0 (9.9–14.1)	
**Age, years**						
**≤65**	92 (60.9%)	13.5 (11.7–15.3)	0.031	100 (65.8%)	11.6 (9.8–13.4)	0.864
**>65**	59 (39.1%)	10.9 (8.4–13.4)		52 (34.2%)	13.3 (11.4–15.4)	
**Sex**						
**Male**	98 (64.9%)	12.3 (10.2–14.3)	0.697	92 (60.5%)	11 (8.2–13.8)	0.044
**Female**	53 (35.1%)	13.3 (9.5–17.1)		60 (39.5%)	13.7 (10.9–16.5)	
**Cachexia**						
**yes**	53 (35.1%)	9.9 (8.4–11.4)	0.0006	59 (38.8%)	9.1 (6.9–11.2)	0.005
**no**	98 (64.9%)	14.3 (12.4–16.2)		93 (61.2%)	14.2 (12.7–15.7)	

*p-values were calculated with Log-rank test.

*OS: Overall survival; mo, months.*

In the first cohort there were 147 deaths (event rate of 97.4%), while four patients were alive without progression while in the validation cohort, there were 144 deaths (event rate of 94.7%) and eight patients were alive without progression at last contact (January 2014), with a median follow-up for living patients of 31.4 months (range, 28.4–32.7). Median OS in the first and second cohorts were 12.5 months (95% CI: 10.9–14.1) and 12.0 months (95% CI: 9.9–14.1), respectively.

In the first and second cohorts of patients OS was significantly shorter among patients with an age of over 65 years and male gender (*p* = 0.031 and *p* = 0.044), respectively, whereas other patient characteristics were not associated with outcome.

**Figure 1:**
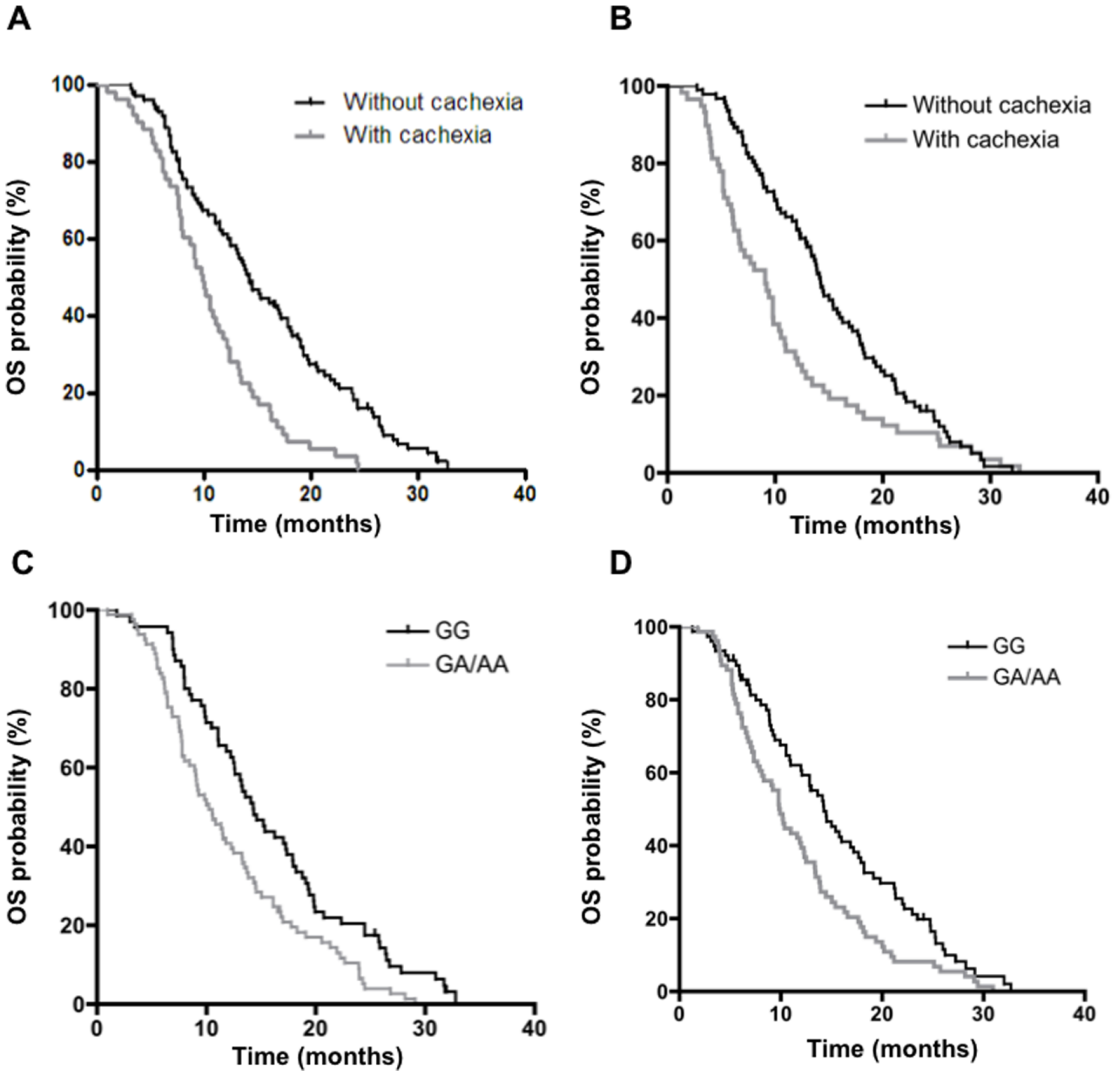
Clinical outcome according to cachexia and *AKT1-rs1130233* polymorphism. (**A** and **B**) Kaplan–Meier survival curves according to cachexia in the first and second cohort of patients. (**C** and **D**) Kaplan–Meier survival curves according to the *AKT1-rs1130233* polymorphism in the first and second cohort of patients. *p*-values were calculated with the log-rank test. OS: Overall survival.

Patients in the validation cohort had a comparable distribution of weight loss, and baseline demographic characteristics were also quite similar between the two cohorts. Importantly, cachexia was significantly associated with shorter OS in both cohorts (*p* = 0.00006 and *p* = 0.005, respectively). The first cohort included 53 cachectic patients (35.1%), with a median OS of 9.9 months (95%CI: 8.4–11.4), compared to 14.3 months (95%CI: 12.4–16.2) of non-cachectic patients, while the validation cohort included 59 cachectic patients (38.8%), with a median OS of 9.1 months (95%CI: 6.9–11.2), compared to 14.2 months (95%CI: 12.7–15.7) of non-cachectic patients (Figures 1A–B).

### Polymorphisms and cachexia

To investigate whether there is an association between cachexia and *IL6-rs1800796*, *AKT1-rs1130233* and *SELP-rs6136* polymorphisms, we performed genotyping using genomic DNA extracted from peripheral blood samples.

Previous studies showed no differences in polymorphisms analyzed in tumors and normal tissues [26], [27]. However, since high level gene amplification in tumor cells might result in homozygous genotypes in individuals who are heterozygous in the germline, we performed preliminary studies of *AKT1* polymorphisms in 45 paired samples of germ-line and cancer DNA, showing identical interindividual genotypes between normal and malignant tissues [19]. Therefore, for the patients enrolled in this study the genotyping was performed in DNA extracted from the available blood samples. Genotyping was successfully carried out in all the DNA samples, and no discrepancies were found in the samples analyzed in duplicate (approximately 10%).

In the first cohort of patients the wild-type *SELP-rs6136* genotype (*AA*) had a frequency of 46.4%, whereas the *AC* and *CC* genotypes were found in 43.7% and 9.9% of the patients, respectively. For the *IL6-rs1800796* polymorphism, the frequencies of the *GG*, *GC* and *CC* genotypes were 59.3%, 37.4% and 3.3%, respectively. Regarding the *AKT1-rs1130233* polymorphism, the *GG*, *GA* and *AA* variants were observed in 52.8%, 39.3% and 7.9% of the cases, respectively. Similar results were observed in the second cohort of patients, as reported in the Table 2. Indeed, no significant differences were measured both the baseline demographic characteristics and in the frequencies of the candidate polymorphic genotypes in the two cohorts of PDAC patients.

**Table 2:** Correlation between cachexia and candidate *SELP*, *AKT1* and *IL-6* SNPs.

			First cohort		Second cohort	
SNP	Cachexia	Genotype	Patients *n* (%)	*p*-value*	Patients *n* (%)	*p*-value*
*SELP rs6136*	**Yes**	**AA**	36 (23.8%)	0.011	36 (23.8%)	0.045
		**AC/CC**	17 (11.2%)		23 (15.1%)	
	**No**	**AA**	45 (29.8%)		40 (26.3%)	
		**AC/CC**	53 (35.2%)		53 (34.8%)	
*AKT1 rs1130233*	**Yes**	**GG**	16 (10.1%)	0.004	21 (13.8%)	0.019
		**GA/AA**	37 (24.5%)		36 (23.7%)	
	**No**	**GG**	54 (35.7%)		53 (34.9%)	
		**GA/AA**	44 (29.7%)		40 (27.6%)	
*IL-6 rs1800796*	**Yes**	**GG**	17 (11.2%)	0.169	18 (11.8%)	0.486
		**GC/CC**	36 (23.4%)		41 (27%)	
	**No**	**GG**	43 (28.5%)		34 (22.4%)	
		**GC/CC**	55 (36.9%)		59 (38.8%)	

*p-values were calculated with Fisher's exact test.

*SNPs, single nucleotide polymorphisms.*

Note: *SELP* and *IL6* SNPs was detectable in all the samples, while the *AKT1* genotype could not be determined in 2 of the patients of the second cohort.

All the polymorphisms followed Hardy–Weinberg equilibrium, as calculated with the *SNP analyzer software* (http://snp.istech21.com/snpanalyzer/2.0/, Table S1) and their allelic frequencies were comparable with those reported in Caucasian populations, and in NCBI and NCI-SNP500 databases. No significant correlations were detected between genotype and baseline demographic characteristics (i.e. age and sex, data not shown).

By grouping patients with *IL6-rs1800796*-*GG versus* those with *GC* or *CC* genotypes we did not find an association with cachexia (Table 2). Conversely, a correlation was observed between the *AKT1-rs1130233* genotype and cachexia, with significantly higher proportion of patients harboring at least one *A*-allele having cachexia (i.e. 37 out of 81, *versus* 16 out of 70 *GG* patients, *p* = 0.004). As shown in Table 2, the *SELP-rs6136* polymorphism was also significantly (*p* = 0.011) associated with cachexia. In particular, 44% of the patients harboring the *SELP-rs6136*-*AA* variants experienced cachexia, compared to 24% of the patients harboring the *SELP-rs6136*-*AC/CC* genotype.

Remarkably, we observed similar results in the validation cohort of patients, showing that individuals who carry the *AKT1-rs1130233-GG* genotype or the *C*-allele of the *SELP-rs6136* polymorphism were at reduced risk of developing cachexia (Table 2). These results were confirmed by unconditional logistic regression, which calculated ORs and their 95%CI for association with cachexia phenotype of each individual SNP, as reported in Table S2.

### Polymorphisms and outcome

Considering that cachexia is a key determinant of cancer-related death, we hypothesized that the candidate polymorphisms associated with cachexia might also affect the clinical outcome.

At univariate analysis, the *AKT1-rs1130233* polymorphism was associated with significantly differential OS. In particular, significantly longer survival was observed in patients harboring the *GG* genotype (14.3 months; 95% CI: 12.1–16.5), in comparison with patients carrying the *GA*/*AA* genotype, who had a median OS of 10.3 months (95% CI: 8.1–12.4; *p* = 0.002; Figure 1C). Similar results were observed in the validation cohort: patients with the *GA* and *AA* genotypes had a worse prognosis (median OS 9.9 months, 95% CI: 8.6–11.1), compared to the patients harbouring the *GG* genotype (14.3 months, 95% CI: 11.7–16.8; *p* = 0.004; Table 3; Figure 1D). To keep at minimum the probability to find a statistically significant difference purely by chance, the usual nominal level (*p* = 0.05) has been lowered to 0.017 by Bonferroni adjustment for multiple comparisons. After the Bonferroni adjustment, the *AKT1-rs1130233* SNP was still significantly correlated to OS. In contrast, *SELP-rs6136* and *IL6-rs1800796* polymorphisms were not associated with the outcome, as reported in Table 3.

**Table 3:** Clinical outcome according to candidate *SELP*, *AKT1* and *IL-6* SNPs.

		First cohort		Second/validation cohort	
SNP	Genotype	OS median mo. (95% CI)	*p*-value	OS median mo. (95% CI)	*p*-value
***SELP rs6136***	**AA**	11.4 (8.4–14.4)	0.665	11.0 (9.1–12.9)	0.519
	**AC/CC**	12.7 (10.1–15.2)		13.7 (10.2–17.1)	
***AKT1 rs1130233***	**GG**	14.3 (12.1–16.5)	0.002	14.3 (11.7–16.8)	0.004
	**GA/AA**	10.3 (8.1–12.4)		9.9 (8.6–11.1)	
***IL-6 rs1800796***	**GG**	11.5 (7.1–16.0)	0.259	11.6 (9.1–14.2)	0.651
	**GC/CC**	12.5 (10.8–14.3)		12.5 (9.7–15.3)	

*OS: overall survival; mo: months.*

In the multivariate analysis, cachexia and age >65 were significantly associated with increased risk of death (Table 4). The Cox proportional hazard regression model also showed the prognostic significance of the *AKT1-rs1130233* polymorphism. In particular, the *GA*/*AA* genotype emerged as a significant predictor for shorter survival, with an increased risk of death of 1.7 (95% CI: 1.2–2.4; *p* = 0.002) and 1.6 (95% CI: 1.2–2.3; p = 0.004), in the first and second cohorts, respectively.

**Table 4:** Factors associated with overall survival in the multivariate analysis.

		First cohort			Second/validation cohort		
Covariates for OS		HR (95%CI)	df	*p*-value	HR (95%CI)	df	*p*-value
**Age, years**	**≤65**	1 (ref)	1	0.032	1 (ref)	1	0.864
	**>65**	1.4 (1.0–2.0)			1.0 (0.7–1.4)		
**Sex**	**Male**	1.1 (0.8–1.5)	1	0.698	1.4 (1.0–1.9)	1	0.046
	**Female**	1 (ref)			1 (ref)		
**Cachexia**	**Yes**	2.2 (1.6–3.2)	1	0.0001	1.6 (1.1–2.3)	1	0.006
	**No**	1 (ref)			1 (ref)		
***AKT1 rs1130233***	***GG***	1 (ref)	1	0.002	1 (ref)	1	0.004
	***GA/AA***	1.7 (1.2–2.4)			1.6 (1.2–2.3)		

*df: degrees of freedom; HR: hazard ratio; OS: overall survival.*

### *AKT1-rs1130233* and expression of Akt1 and phospho-Akt1 in skeletal muscle

**Figure 2:**
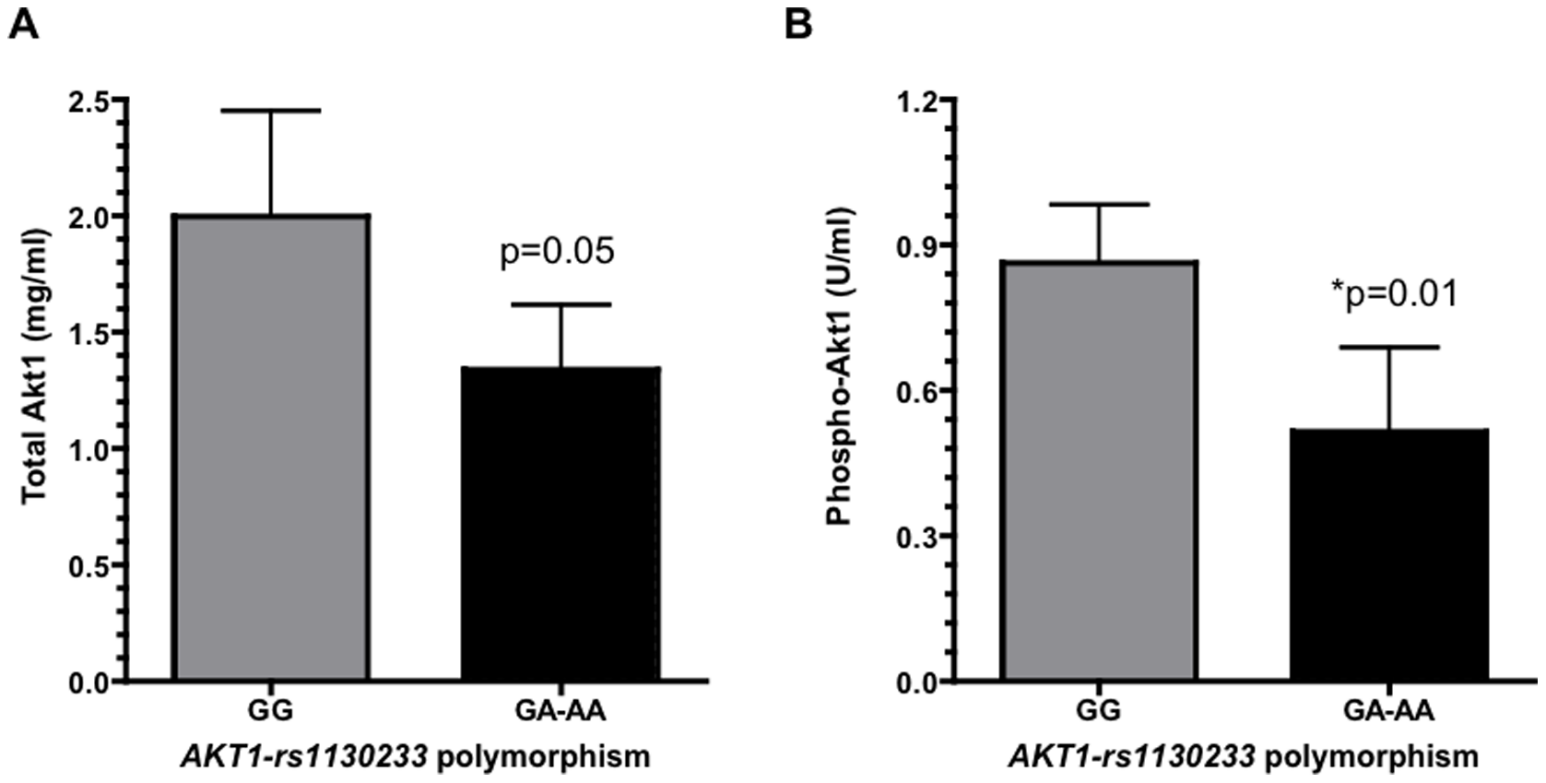
Akt1 expression in muscle samples according to the *AKT1-rs1130233* polymorphism. Bar graphs illustrating the mean±SD expression of total Akt1 (**A**) and phospho-Akt1 (**B**) in muscle samples from patients with differential *AKT1-rs1130233* genotypes (N = 9 samples in each group) *p<0.05.

The significant association of the *AKT1-rs1130233* polymorphism with cachexia and clinical outcome prompted us to perform an exploratory study on its role on the level and activity of Akt1 in a panel of muscle biopsies. As illustrated in Figure 2, the samples with the *GA* or *AA* genotypes had a trend toward a significantly lower protein expression (approximately −30%) of Akt1 in comparison to the samples with *AKT1-rs1130233-GG* genotype (*p* = 0.050). These results were similar to the reduction in phospho-Akt1 levels, resulting in a significant lower average level of phosphorylation at Ser437 of Akt1 in the *GA/AA* samples compared to the *GG* samples (*p* = 0.006). However, when we normalized the phospho-Akt1 levels to the total-Akt1 levels in each sample we observed that in all except one case, the samples with *GA/AA* genotype had a lower ratio between phospho-Akt1 and total Akt1 vs. the samples with the *GG* genotype (Fig. S1, *p* = 0.003). These data suggest that the *AKT1-rs1130233*-*GA/AA* genotypes might confer a reduced activity to Akt1, and thus reduce the antiapoptotic activity of this pivotal regulator of apoptotic signaling.

## Discussion

In the present study we demonstrated the importance of *AKT1-rs1130233* polymorphism as a predictive marker of risk of cachexia and death in patients with locally-advanced or metastatic PDAC. Our pharmacogenetic analysis also identified an association between cachexia and the *SELP-rs6136-AA* genotype, in agreement with a previous study in 120 PDAC patients [16].

Conversely, no statistically significant correlation was detected for the *G-*allele of the *rs1800796* polymorphism in the *IL6* gene, which was associated with increased susceptibility to cachexia and decreased survival time of stage II and III Chinese PDAC patients [14]. These discrepancies could suggest that pharmacogenetic associations are not always reproducible in studies in populations with different ethnic backgrounds, as well as in different clinical stages. However, the minor allele frequencies, of approximately 4%, in our cohorts, greatly limited the statistical power for the analysis of this polymorphism. Larger studies with homogeneous settings of patients are essential to investigate the role of emerging biomarkers before planning of prospective trials.

PDAC patients have the highest risk of developing cachexia among the gastrointestinal tumors, and it has been shown that cachexia is correlated with poor prognosis, reduced treatment tolerance and a significant reduction in the quality of life of these patients [28], [29].

Cachexia can be caused by a complex interplay of mechanisms including medical problems such as diabetes, tumor stage, and duodenal or common bile duct obstruction, which cause pain, nausea, dysphagia, gastroparesis, pancreatic insufficiency and malabsorption [30]. Recent studies have shown that also neural invasion, which commonly occurs in PDAC, is related to cachexia. Nerve damage from PDAC can indeed activate astrocytes, which subsequently induce lipolysis and muscle atrophy [31]. Furthermore, the increase in the sympathetic nervous system activity might cause lipolysis in adipose tissue and muscle atrophy [32]. In addition to these mechanical factors, several other mechanisms have been proposed to drive the pathophysiology of PDAC cachexia and there is evidence that anorexia and hypercatabolism can be triggered by cytokines, circulating hormones, neuropeptides, neurotransmitters, and tumor-derived factors.

Several studies showed that increased levels of cytokines, such as IL-6, were associated with weight loss and poor prognosis in PDAC patients [8]–[10]. However, the variable predisposition to cachexia may be also due to the patient's genotype, and a comprehensive pharmacogenetic study demonstrated the association of cachexia with the *rs6136* polymorphism of the gene *SELP*. This gene encodes the cell adhesion protein P-selectin, which was found to be upregulated in murine and rats models of cachexia caused by both acute and chronic inflammatory insults [16]. These data revealed that P-selectin has a relevant role in both animal models and in cachectic cancer patients. However, no data are yet available on its role as a risk factor or as a potential mediator of the cachectic process.

Since apoptosis is reported to take place in wasting muscle in cachexia, several other studies evaluated the key role of Akt1 in developing cancer cachexia [33]–[35]. Akt1 is a serine/threonine kinase acting as a critical mediator of growth factor-induced survival. Survival factors can suppress apoptosis in a transcription-independent manner by activating Akt1, which then phosphorylates and inactivates components of the apoptotic machinery [32], [36]. Moreover, in skeletal muscle, Akt1 plays a very central role in the control of both muscle protein synthesis, via mTOR, and protein degradation, via the transcription factors of the FoxO family. This suggests a pivotal role in excessive loss of muscle mass associated with several diseases, including myopathies and muscular dystrophies, as well as in cachexia associated with systemic disorders such as cancer, diabetes, sepsis and heart failure [13]. Schmitt and colleagues demonstrated a cachexia-associated loss of Akt-dependent signaling in human skeletal muscle of cachectic patients compared to non-cachectic patients, using muscle biopsies from 16 PDAC patients undergoing pancreatectomy [33]. Notably, *AKT1* is a highly polymorphic gene, and functional SNPs might affect Akt1 levels and influence apoptosis induction [21].

Therefore, in the current study we investigated the association with cachexia and the prognostic value of the candidate functional polymorphisms *IL6-rs1800796*, *AKT1-rs1130233* and *SELP-rs6136* in a first cohort of 151 patients with locally-advanced or metastatic PDAC. Then we validated the results by replicating the association study in an independently recruited group of 152 patients.

To the best of our knowledge this is the first study demostrating that individuals who carried the *A*-allele for *AKT1-rs1130233* polymorphism were at increased risk of developing cachexia. Moreover, these patients survived significantly shorter, compared to patients carrying the *GG* genotype. Remarkably, the Cox proportional hazards regression model used for the multivariate analysis illustrated the independent prognostic value of *AKT1-rs1130233*.

Of note, the *AKT1-rs1130233* is a synonymous polymorphism, i.e. a polymorphism where the change in the base in the DNA sequence does not alter the amino acid encoded due to the redundancy of the genetic code. Because synonymous SNPs do not change the composition of the protein product, they have largely been assumed to exert no discernible effect on gene function or phenotype. However, studies on artificial site-directed silent mutagenesis of synonymous codons in several genes support the hypothesis that altered translation kinetics of mRNA, caused by altered translation kinetics and folding, might affect final protein conformation [37]. Moreover, Kimchi-Sarfaty and collaborators demonstrated that the P-glycoprotein inhibitors cyclosporin and verapamil were less effective against proteins that were produced from polymorphic haplotypes that did not change the amino acid sequence but slow down the ribosome traffic at the corresponding mRNA regions [38]. These alterations may thus affect the cotranslational folding pathway, resulting in a different final conformation and function. Although we could not test this hypothesis because of the lack of conformation-sensitive monoclonal antibodies for Akt1, previous studies in different tissue types showed that the *AKT1-rs1130233-AA* variant correlated with lower *AKT1* mRNA expression [19], and with lower protein levels, contributing to lower apoptotic response [21].

To gain further insight into the mechanisms behind our findings, we performed additional studies showing a significant association of Akt1 phosphorylation status in muscle biopsies and the *AKT1-rs1130233* polymorphism. In particular we observed a significant reduction of the phosphorylation at Ser437 of Akt1 in the *GA*/*AA* samples compared to the *GG* samples. These results suggest that the *AKT1-rs1130233-GA*/*AA* genotypes might reduce the activity to Akt1 and favour the induction of apoptosis, which in turn causes muscle atrophy and increases cachexia, in agreement with the clinical results.

Remarkably, a recent study by He and colleagues showed that tumor-secreted microvesicles contain an elevated expression of microRNA-21 (miR-21) and induce myoblast apoptosis in cancer cachexia via a Toll-like receptor 7-c-Jun N-terminal kinase-dependent pathway [39]. We have recently shown that miR-21 is up-regulated and acts as an oncogene in pancreatic intraductal papillary mucinous neoplasms and PDAC [40], [41]. Moreover, we demonstrated that modulation of Akt1 phosphorylation and apoptosis induction may contribute to the prognostic role of miR-21, as well as in gemcitabine chemoresistance [42]. Indeed, among the multiple targets of miR-21 in PDAC we showed the key role of PTEN, leading to *AKT1* regulation. Therefore, we might hypothesize that the different *AKT1* genotypes might affect its inhibition by PTEN after stimulation by miR-21, favouring tumor-induced muscle wasting through apoptosis induction.

However, previous studies reported controversial relationship between miR-21 expression and PTEN regulation, both in the preclinical and clinical setting [43], as well as on the functional role of candidate *AKT1* SNPs [44]–[46]. More genotype-phenotype correlation studies and functional analyses of other critical genes involved in the Akt1 pathway are warranted. Further research to elucidate the intricate mechanisms involved in the induction and maintenance of PDAC cachexia, should aid in the development of future therapeutic targets. In particular, it remains to be determined if modulation of phospho-Akt by specific drugs might alter the development of cachexia. Akt1 might indeed be a candidate therapeutic target in cancer cachexia and even survival of PDAC, after the selection of the patients according to their genotype.

A major strength of the present study is that it was carried out in a homogeneous setting of patients with pancreatic cancer. The results of multivariate analysis indicate the noteworthiness of the prognostic role of *AKT1-rs1130233*. Moreover, the minor allele frequency of this polymorphism in a random Caucasian population is frequent (i.e., 28% according to the SNP-NCBI cancer database). Thus, these findings might be relevant to a large number of patients. Conversely, the main limitations of this study include the retrospective explorative study design and the lack of prospective randomized studies on the potential predictive role of *AKT1-rs1130233* for chemotherapy activity.

## Conclusion and Future Perspective

*AKT1-rs1130233* and *SELP-rs6136* polymorphisms emerged as a predictive risk factor of developing cachexia in locally-advanced and metastatic PDAC. Moreover *AKT1* polymorphisms may play a prognostic role. Since pancreatic cancer is such a lethal disease, any biomarker that can help to better stratify patients for developing cachexia might have crucial clinical applications. Ultimately, validation of the value of the emerging candidate polymorphisms in future prospective trials will offer new tools to improve the clinical management of advanced PDAC patients.

## Supporting Information

Figure S1 **Phospho/Total Akt1 expression in muscle samples according to the** ***AKT1-rs1130233*** **polymorphism.** Bar graphs illustrating the mean±SD expression of the ratio of total Akt1 and phospho-Akt1 in muscle samples from patients with differential *AKT1-rs1130233* genotypes. *p<0.05.
(PPT)

Table S1 **Genotyping of PDAC patients for the candidate** ***SELP*** **,** ***AKT1*** **and** ***IL-6*** **SNPs.**
(DOC)

Table S2 **Logistic regression analysis of cachexia and** ***SELP*** **,** ***AKT1*** **and** ***IL-6*** **SNPs.**
(DOC)
