# Supplementary figures and images for: AKT1 and SELP Polymorphisms Predict the Risk of Developing Cachexia in Pancreatic Cancer Patients
Source: PLoS One. 2014 Sep 19;9(9):e108057. doi: 10.1371/journal.pone.0108057 (PMC4169595; doi:10.1371/journal.pone.0108057)

## Slide 1
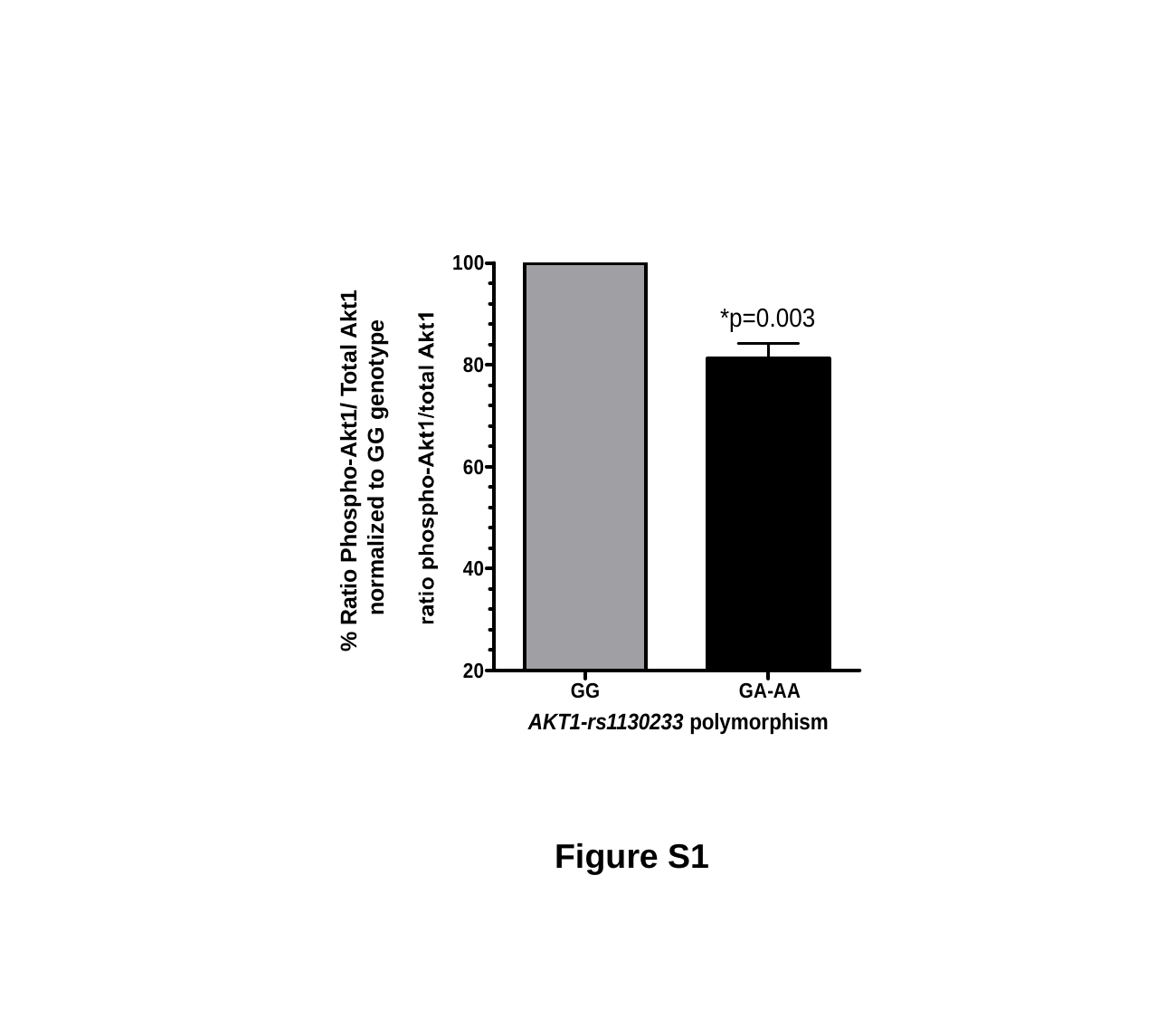

% Ratio Phospho-Akt1/ Total Akt1
normalized to GG genotype
Figure S1

Supplement: Figure S1 — Phospho/Total Akt1 expression in muscle samples according to the AKT1-rs1130233 polymorphism. Bar graphs illustrating the mean±SD expression of the ratio of total Akt1 and phospho-Akt1 in muscle samples from patients with differential AKT1-rs1130233 genotypes. *p<0.05. (PPT) [file pone.0108057.s001.ppt]
