# Supplementary material for: AKT1 and SELP Polymorphisms Predict the Risk of Developing Cachexia in Pancreatic Cancer Patients
Source: PLoS One. 2014 Sep 19;9(9):e108057. doi: 10.1371/journal.pone.0108057 (PMC4169595; doi:10.1371/journal.pone.0108057)
Supplement: Table S1 — Genotyping of PDAC patients for the candidate SELP , AKT1 and IL-6 SNPs. (DOC) [file pone.0108057.s002.doc]

**Supplemental Table S1**

| **Table S1.** Genotyping of PDAC patients for the candidate *SELP*, *AKT1* and *IL-6* SNPs | | | | | |
| --- | --- | --- | --- | --- | --- |
|  | **First cohort** | | | **Second/validation cohort** | |
| **SNP** |  | **Patients**  **n (%)** | **HWE**  ***p*-value** | **Patients**  **n (%)** | **HWE**  ***p*-value** |
| ***SELP-rs6136*** |  |  |  |  |  |
|  | *AA* | 81 (53.6) |  | 76 (50.0) |  |
|  | *AC* | 60 (39.7) |  | 66 (43.4) |  |
|  | *CC* | 10 (6.6) | 0.911 | 10 (6.6) | 0.859 |
| ***AKT1-rs1130233*** |  |  |  |  |  |
|  | *GG* | 70 (46.4) |  | 74 (49.3) |  |
|  | *GA* | 65 (43.1) |  | 64 (42.7) |  |
|  | *AA* | 16 (10.6) | 0.980 | 12 (8.0) | 0.972 |
| ***IL6-rs1800796*** |  |  |  |  |  |
|  | *GG* | 91 (60.3) |  | 100 (65.8) |  |
|  | *GC* | 51 (33.8) |  | 44 (29.0) |  |
|  | *CC* | 9 (6.0) | 0.923 | 8 (5.3) | 0.527 |
| *HWE: Hardy–Weinberg equilibrium* | | | | | |
