# Supplementary material for: AKT1 and SELP Polymorphisms Predict the Risk of Developing Cachexia in Pancreatic Cancer Patients
Source: PLoS One. 2014 Sep 19;9(9):e108057. doi: 10.1371/journal.pone.0108057 (PMC4169595; doi:10.1371/journal.pone.0108057)
Supplement: Table S2 — Logistic regression analysis of cachexia and SELP , AKT1 and IL-6 SNPs. (DOC) [file pone.0108057.s003.doc]

**Supplemental Table S2**

| **Table S2.**  Logistic regression analysis of cachexia and *SELP*, *AKT1* and *IL-6* SNPs | | | | | | |
| --- | --- | --- | --- | --- | --- | --- |
|  | | | **First cohort** | | **Second cohort** | |
| **SNP** | **Risk genotype** | **df** | **OR (95%CI)** | ***p*-value** | **OR (95%CI)** | ***p*-value** |
| ***SELP-rs6136*** |  |  |  |  |  |  |
|  | *AA* | 1 | 2.4 (1.1-5.0) | 0.017 | 2.0 (1.0-4.0) | 0.039 |
| ***AKT1-rs1130233*** |  |  |  |  |  |  |
|  | *GA-AA* | 1 | 3.0 (1.4-6.3) | 0.003 | 2.1 (1.1-4.1) | 0.035 |
| ***IL6-rs1800796*** |  |  |  |  |  |  |
|  | *GG* | 1 | 0.6 (0.3-1.2) | 0.139 | 0.7 (0.4-1.5) | 0.416 |
| *df: degree od freedom; OR: odds ratio; SNPs: single nucleotide polymorphisms* | | | | | | |
